# Soil storage and growth media shape culture-based recovery of pyrophilous bacteria and fungi from burned soils

**DOI:** 10.1371/journal.pone.0359320

**Published:** 2026-11-13

**Authors:** Dylan J. Enright, Aishwarya Veerabahu, Ryan J. Quaal, Marely Vega, Anna Nguyen, Jenna Grindeland, James W. J. Randolph, Maria E. Ordoñez, Sydney I. Glassman

**Affiliations:** Department of Microbiology and Plant Pathology, University of California Riverside, Riverside, California, United States of America; Universidad Autonoma de Chihuahua, MEXICO

## Abstract

Increasingly frequent and severe fires worldwide underscore the need to understand how pyrophilous microbes survive, thrive, and drive post-fire ecosystem regeneration. However, characterizing their traits through omics and phenotypic assays is limited by the lack of cultured isolates. Here, we collected burned soils after a severe California shrubland wildfire and used a culturomics approach to test how culture media type and soil storage method affected the abundance and diversity of pyrophilous bacteria and fungi. Using fresh soils, we compared three media types: rich media (Lysogeny Broth [LB] for bacteria; Malt Yeast Agar [MYA] for fungi), oligotrophic Reasoner’s 2A (R2A), and a newly developed pyrogenic organic matter (PyOM) media. We also evaluated four soil storage methods using rich media: air-drying, refrigeration at 4°C, and freezing at –80°C with or without glycerol. R2A recovered the greatest richness of bacterial and fungal genera, although PyOM medium isolated several taxa not recovered on other media. For bacteria, freezing soils at –80°C without glycerol preserved the greatest genus richness, whereas freezing with glycerol preserved the greatest species richness. For fungi, freezing at –80°C without glycerol preserved the most species while maintaining genus richness comparable to fresh and refrigerated soils. These findings guided the development of a culture collection of more than 500 pyrophilous bacterial and fungal isolates. Lessons learned from media type and soil storage can also be applied to the cultivation of environmental soil microbes from many habitat types and improve culture collections of fastidious microbes.

## Introduction

Although advances in the fields of molecular biology and DNA sequencing have freed microbiologists from working with culturable species exclusively [1], few laboratory resources are as valuable as live cultures. Pure microbial cultures are essential for high quality whole-genome assembly [2,3], genetic engineering [4], and phenotypic profiling [5–9]. Additionally, microbial isolates greatly improve the interpretation of metabolomics data [10], and possession of a diverse culture collection can unlock a plethora of microbiological applications [4,11]. Once isolated and properly stocked, culture collections become a permanent resource for future microbiological research and generate new avenues for collaborative research [12–15]. However, there are many hurdles to creating and maintaining microbial culture collections and each collection has unique challenges [12,15].

The factors that can be modified to culture microorganisms are nearly as diverse as the microbes themselves. For example, media selection requires consideration of numerous variables including proper carbon sources, nitrogen and phosphorus content, salt concentrations, pH, and micronutrients [11,16,17]. Further abiotic variables to consider include incubation temperature and duration, water and oxygen availability, and whether to alternate light and dark cycles or to make the media nutrient rich or poor (oligotrophic) [17,18]. This complexity contributes to many microorganisms remaining uncultured [19]. Though recent advances have begun to make the adage of “99% of microbes are unculturable” outdated [20], many microbes remain uncultivated, and creating diverse culture collections, particularly for fastidious microbes, remains challenging.

One group of understudied microorganisms that is of growing interest are the pyrophilous (or “fire-loving”) microbes. Pyrophilous microbes, that massively increase in abundance post-fire but were rare or absent pre-fire [21,22], may persist for many years post-fire [23], and greatly influence post-fire ecosystem recovery [24,25]. Wildfires dramatically change landscapes and create legacy effects that can impact soil microorganisms and ecosystems for many years [26–28]. While knowledge on distribution and abundance of pyrophilous microbes is expanding, information on their traits remains limited [7,21], despite global increases in frequency and intensity of fires [29]. Cultured isolates are essential for understanding how pyrophilous microbes compete, cycle nutrients, and shape post-fire ecosystems, yet establishing culture collections remains technologically and logistically challenging.

Beyond the challenges inherent to building microbial culture collections, culturing wildfire-associated microbes presents unique logistical obstacles, particularly because the unpredictable nature of wildfires complicates timely sample processing and proper soil storage. Large-scale culturing efforts rely on many things including having personnel available, having consumables ordered, and media and isolation conditions being selected and created. Thus, post-fire soil samples may need to be stored for several weeks to months prior to culturing. Indefinite storage durations is a logistical problem shared among many environmental microbial culturing efforts ranging from marine systems [30] to Antarctic soils [31]. Some insights on overcoming these logistical hurdles have been made, but results are inconsistent, some storage methods may reduce microbial viability, and no comprehensive study of soil storage impacts on fungi exists [12,32,33].

In addition to difficulties surrounding proper soil storage, wildfires also present challenges for proper media selection because they radically alter environmental nutrient and resource landscapes [28]. For example, wildfires reduce soil moisture, increase pH [34], and pyrolyze wood and labile, easily accessible carbon sources into difficult to degrade pyrogenic organic matter (PyOM) [35]. PyOM is often enriched in polycyclic aromatic hydrocarbons (PAHs) that may require specialized metabolic pathways to process [24,25,36], which can lead to accumulated char persisting in the environment for up to and exceeding 100 years [35]. Wildfires also release large amounts of inorganic nitrogen through combustion and ash deposition [26,37], which may alter post-fire microbial nutrient strategies and restrict survival to a subset of microbes. For example, many species of mycorrhizal fungi are nitrophobic [38], preventing them from establishing essential plant associations in high nitrogen environments. These nutrient shifts may result in commonly used laboratory media not providing appropriate resources to cultivate pyrophilous microbes.

To address these challenges, we used soil from a local high severity California chaparral shrubland wildfire to test the best 1) laboratory media and 2) soil storage methods to retain the greatest richness, culturing viability, and most appropriate diversity of pyrophilous bacteria and fungi, with the eventual goal of creating a large culture collection to use to test the traits of pyrophilous microbes. For media type, we tested culturing immediately from fresh soil onto rich (lysogeny broth (LB) agar for bacteria, malt-yeast agar (MYA) for fungi), oligotrophic (Reasoner’s 2 Agar (R2A)), or a media created in-house from PyOM. For soil storage, we tested culturing immediately from fresh soils on rich media versus soils stored for 3 months in four ways (air-dried, refrigerated at 4°C, and frozen at −80°C with and without glycerol). We tested the impact of our treatments on colony forming units (CFUs) and microbial richness and composition (assessed via Sanger sequencing of 16S for bacteria or ITS for fungi; Fig 1). We hypothesized (H1) that other than fresh soil, air-dried soil would retain the greatest abundance and genera richness of pyrophilous bacteria and fungi since burned soils are already hot and dry. We further hypothesized (H2) that rich media would yield the greatest richness and abundance of microbes, but that PyOM media would be necessary to culture some of the most dominant and metabolically unique pyrophilous microbes. Although we specifically sought to create a pyrophilous culture collection, the insights gleaned here can apply to media selection and storage choices to cultivate soil microbes from many soil environments and a wide array of fastidious microbes.

**Fig 1:**
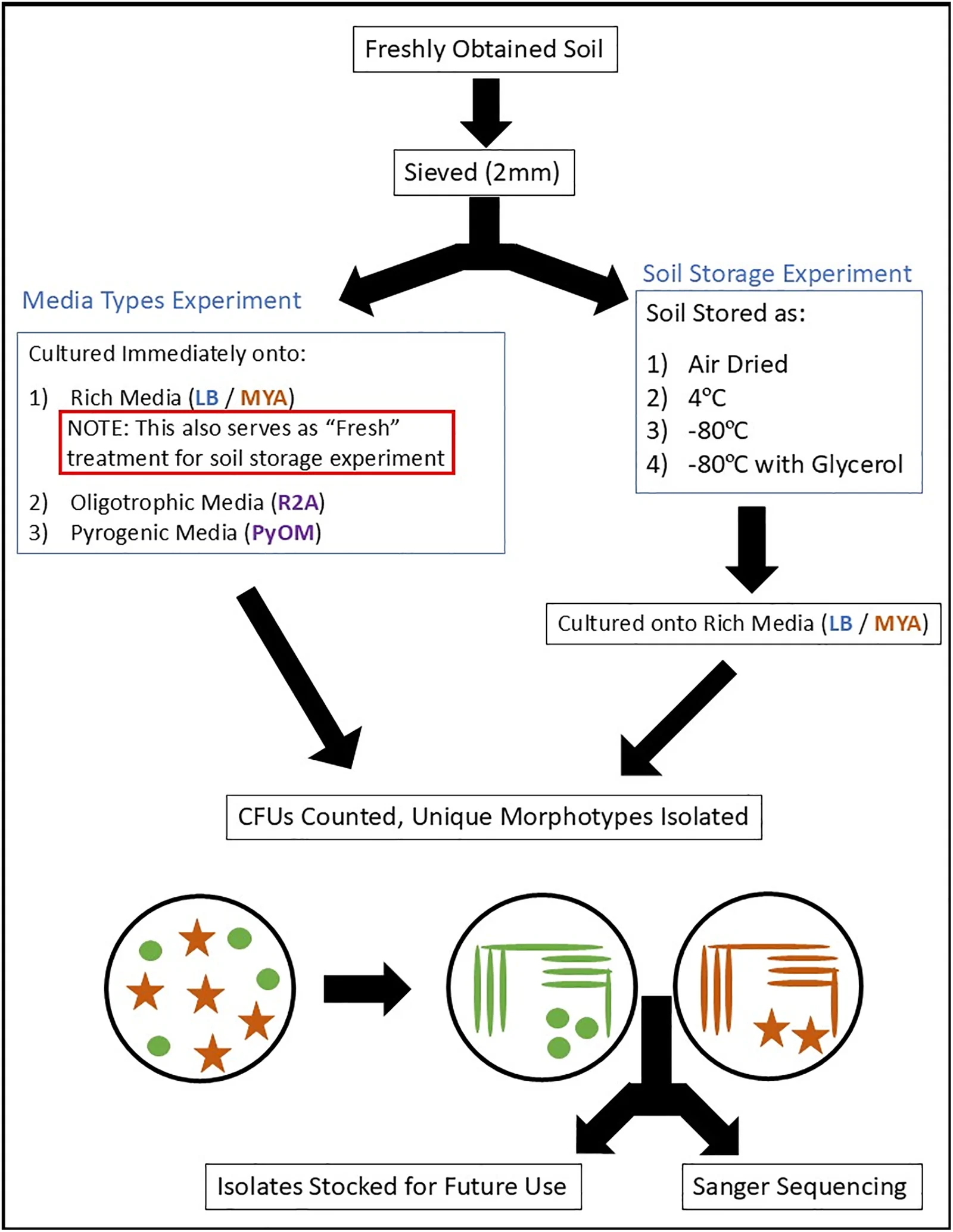
A conceptual diagram of the experimental design used in this experiment. “LB” is abbreviated for Lysogeny Broth, “MYA” is Malt Yeast Agar, “R2A” is Reasoner’s 2 Agar, and “PyOM” is Pyrogenic Organic Matter. Note that the cultures obtained from fresh soil plated onto rich media also serve as the “Fresh” storage condition for the soil storage experiment. Green circles and orange stars symbolize two different morphotypes collected from the same initial plate.

## Materials and methods

### Site description

We sampled soil from The El Dorado wildfire, which burned ~92km^2^ Manzanita (*Arctostaphylos)* dominated chaparral shrublands at high severity, in Yucaipa, CA, ~ 45 minutes from UC Riverside, from September through November of 2020. We used our high temporal resolution Illumina MiSeq sequencing dataset of soil bacterial and fungal communities from a nearby high-severity Manzanita dominated chaparral wildfire [39] to select 6 months post-fire as the ideal timeframe to isolate the greatest diversity of pyrophilous bacteria and fungi because this time point had the largest number of pyrophilous genera relative–other time points. Further, it was after rains had allowed for dispersal or activation of many taxa of pyrophilous microbes. Burned sites (34°05’15.3“N 116°59’52.5”W), with site permission given by the San Bernadino National Forest, were characterized as having 50 to 75 percent slopes with dry, sandy soils in the Springdale family-Lithic Xerorthents association (https://websoilsurvey.nrcs.usda.gov/app/). At the time of collection gravimetric soil moisture was 9.72% (±2.7%) per gram soil.

### Soil collection

Following the same experimental design as our previous study in a nearby chaparral wildfire [39], we established 6 burned plots, each with four 1m² subplots 5m from the center in each of the cardinal directions. For the culturing experiment, in June 2021, at roughly 6 months after the fire was extinguished, we collected soils from 3 of the highest severity burned plots at each of the 4 subplots (12 total soil samples) from 0–10 cm below the ash layer of burned soil. We used ethanol cleaned releasable bulb planters to collect ~250 ml soil per sample, stored them in sterile whirl-pak bags, and processed them at UC Riverside within 24 hours.

### Experimental design and media versus storage selection experiments

After collection, 3g of soil from all 12 subplots was homogenized, 2 mm sieved, and either used as fresh inoculum for cultivation for the media type experiment (described below), or stored in four different ways for the storage experiment: air dried, refrigerated (4ºC), and frozen (−80ºC) with or without glycerol (Fig 1). After 3 months of storage, stored soil samples were cultured onto rich media. Thus, cultures isolated from fresh soils on rich media served as both the “rich media” condition for the media experiment and the “fresh” comparison for the soil storage experiment (Fig 1). For samples stored in glycerol (20%), 1g of fresh soil was mixed with 1mL of glycerol resulting in an approximately 1:1 w/v soil to glycerol slurry prior to freezing.

For each treatment, 1 g of soil was suspended in 5 mL sterile DI water in four replicate Falcon tubes, serially diluted (1:10–1:10,000), and 100 µL of each dilution was plated onto 10 replicate plates targeting either bacteria or fungi. All fungal plates contained ampicillin and gentamycin, while bacterial plates contained cycloheximide. In total, 560 plates were prepared (2 kingdoms × 4 dilutions × 10 replicates × 7 treatments plus sterile controls).

### Culturing medias used

For our media type experiment, we cultured soil dilutions from fresh soil samples onto four different medias: rich (LB for bacteria, MYA for fungi), oligotrophic (R2A), or PyOM media. We produced LB broth by adding 15g of powdered LB Broth (Fisher Scientific, CAS# 73049-73-7) and MYA broth by adding 5g each of powdered Malt Extract (Research Products International, Genessee, CAS# 8002-48-0) and Yeast Extract (Sigma-Aldrich, Cat # 70161) per liter of DI water. For solid agar media, we added 15g per liter of bacteriological grade agar (Apex Chemicals, Cat # 20–273) to either the LB or MYA broth. We produced R2A agar with powdered pre-mixed R2A agar (Criterion, Cat # 16721) following manufacturer directions.

We developed an in-house recipe of PyOM media using biochar harvested from the El Dorado Fire burn sites (S1 Fig in S1 File). To reduce variations in chemical content across char collections, the PyOM media used in this study was derived from a single acquisition of char. Char pieces that were uniformly black in color, brittle, and appeared fully pyrolyzed were selected from the same plots where soil samples were taken. First, we ground biochar into a fine powder with a coffee grinder (Chefman, Model: RJ44-OPP-RED) for 3–5 minutes at maximum speed. Then, we added 50g of ground biochar to a 2L Erlenmeyer flask, added 1200mL of DI water, covered the mouth of the flask with a watchglass, stirred on a hotplate for 24 hours at 40°C, then removed from heat to settle for 8 hours. We then siphoned liquid contents into a clean Erlenmeyer flask, being careful to not disturb the bottom sediment. We passed the liquid through cheesecloth, and then twice through a coffee filter to remove any remaining sediment before transferring to clean containers for autoclaving. We added 13g of bacteriological grade agar (Apex Chemicals, Cat # 20–273) to each liter of PyOM media, since adding 15g resulted in under-dissolved agar in the final product. This resulted in a media that should reflect the more complicated carbon sources found in char (PAHs, benzoic compounds, etc.) and contained almost 10x more nitrate than LB as assessed by GC mass spectroscopy (average [NO_3_^-^] of 0.27 mg/L in LB versus 1.2 mg/L in PyOM).

We used DI water for all media types (LB, MYA, R2A, PyOM) and all were autoclaved at 121ºC for 45 minutes prior to pouring media into petri dishes. Once media cooled to 60ºC after autoclaving, we added antimicrobials to the liquid agar. For media used to isolate fungi and intended to resist bacterial growth, we added filter sterilized gentamycin and ampicillin (Hardy Diagnostics, Santa Maria, CA) to a final concentration of 10 mg/mL each. For media used to isolate bacteria and intended to resist fungal growth, we added filter sterilized cycloheximide (Research Products International, CAS # 66-81-9) to a final concentration of 20 mg/L.

### Impacts measured

Plates were incubated at 25°C for ~1 week, after which colony-forming units (CFUs) and unique morphologies were counted. Plates containing 30–500 colonies per plate were considered countable, yielding 6–9 replicate plates per treatment. Unique bacterial and fungal morphotypes were isolated, DNA extracted, and Sanger sequenced, and isolates stocked (Fig 1). Diversity was assessed using richness of total or pyrophilous genera or taxa captured per treatment.

Microbes were morphotyped by size, color, and growth rate. For bacteria and yeasts, individual colonies were picked with sterile pipet tips and streaked onto fresh LB (without antifungals). For filamentous fungi, a 1 cm² agar square from the colony edge was transferred onto fresh MYA (without antibacterials). When microbes failed to establish on LB or MYA, they were maintained on their original media (R2A or PyOM). Isolates were incubated at 25°C for 1 week and re-isolated three times to obtain pure cultures for DNA extraction and stocking.

### DNA extraction

DNA was extracted from all unique isolates using home-made Extract-N-Amp Extraction Solution made by combining 10 ml of 1M pH 8.0 Tris-HCl, 1.86g KCl, 0.37g EDTA, and 80 mL DI water, then adding 1M NaOH until pH 9.5 and then diluting to a total volume of 100mL and filter sterilizing. For bacteria, one well-isolated colony was picked using a sterile 10 µL pipet tip, and for fungi, a 1mm^2^ area of the growing edge of the fungus was scraped with a flame-sterilized probe. The tip or probe was then submerged and swirled in 10 µL of Extraction solution, which was heated at 65ºC for 10 minutes followed by 95ºC for 10 minutes in a Thermocycler (BioRad). Once finished, 10 µL of 3% BSA was added, incubated overnight at 4ºC, then PCR amplified or frozen at −20ºC.

### PCR and sanger sequencing

The bacterial 16S rRNA region was amplified using the PA-PH primer pair [40] and the fungal ITS region was amplified with the ITS1F forward [41] and ITS4 reverse primer [42]. Each 20 µl PCR reaction contained 13.64 µl of molecular grade water, 0.16µL of Taq polymerase (New England Biolabs, Ipswitch MA) and 2µL of 10x Taq Polymerase Buffer containing MgCl_2,_ 2µL of 10x solution of equally mixed dNTPs, 0.6µL of each primer at 10μM, and 1µL of DNA. Thermocycler conditions were as follows: 94℃ for 2 minutes, then 35 cycles of 57℃ (for fungi) or 62℃ (for bacteria) for 1 minute, 72℃ for 1 minute, then 94℃ for 1 minute, ending with a final extension of 72℃ for 8 minutes [43]. We cleaned DNA with 3µL of diluted Exosap mastermix (Applied Biosystems, Waltham MA) per 7µL PCR product before diluting and preparing for Sanger sequencing. Samples were Sanger sequenced in the forward direction at the UCR Institute for Integrative Genome Biology Genomics Core services.

### BLAST identification

Raw sequences as .ab1 chromatogram files were trimmed in either SnapGene or Geneious Prime using the same parameters to remove low quality regions (Phred scores < 30, preserving the largest continuous region of good quality sequence) then saved as FASTA files. Taxa were identified using NCBI BLASTn by generating hit tables of the top 10 matches, with IDs assigned based on query cover, e-value, percent identity, and consensus among hits of similar quality. Isolates were identified to species if query cover was 100%, e-value < 0.01, and percent identity >98%; to genus at ~95%, family at 90%, class at 85–90%, and phylum at <85%. Isolate names were preserved from the best-matching BLAST identification.

However, given the difficulty of identifying species with only rRNA fragments, genus level identification is the most reliable and species identifications reflect our best estimation of taxon identity. Taxonomic identifications were used to estimate richness and composition for each treatment. Sanger sequences are accessioned at NCBI Genbank under accession numbers PV662515 - PV662757 for 16S and PV620023 – PV620259 for ITS.

### Creation of microbial inventory

Once isolates were morphotyped and identified, we gave them unique identifiers, and we stocked them for long-term storage to add to our pyrophilous culture collection. Bacterial isolates were grown in LB broth (3 mL) shaken at 180 rpm for 24–72 hrs until turbid, then mixed 1:1 with 40% sterile glycerol (final glycerol concentration of 20%) in cryovials and stored at −80 °C. Fungal isolates were grown to within 1 cm of the plate edge, then 5 mm agar plugs from the colony margin were transferred into screw-cap tubes containing 1 mL autoclaved tap water [44]. Duplicate stock tubes were prepared for each isolate.

### Statistical analysis

All statistical analyses and figures were produced in R 4.0.2 and all scripts are available at: https://github.com/DylanEnright93/Culturing_Pyrophilous_Microbes.

Normality was tested with a Shapiro test, then a two-way ANOVA was used to test the effect of soil storage or media treatment on bacterial and fungal CFU counts, followed by a post-hoc Tukey HSD test. Because Sanger identified only unique morphotypes, we have estimates of total richness of species and genera per treatment, but we do not have replicates for statistical analysis. We identified isolates as pyrophilous based on literature support [21,22,39,45–50] and compared the percent of total fungal or pyrophilous fungal isolates obtained per treatment.

We created heatmap-like visualization of genera and species level diversity with the ggplot2 function “geom_tile” [51].

### Ethics statement

This study did not involve human participants, human tissue, or vertebrate animals. Therefore, institutional ethics approval and informed consent were not required. Soil samples were collected from publicly accessible or appropriately permitted locations, and all field sampling and research activities were conducted in accordance with applicable regulations and permissions.

## Results

### Microbes obtained in media type and soil storage experiment

We isolated a visually diverse array of bacterial and fungal morphotypes from our soil storage and media selection experiments (Fig 2) resulting in 170 bacterial and 160 fungal isolates (S1 Table) of which we characterized 138 bacterial (86.3%) and 152 fungal (95%) as pyrophilous based on the published literature (S2 Table). The 170 total bacterial isolates represented 4 phyla, 10 orders, 31 genera, and 83 taxa. The total 160 fungal isolates represented 3 phyla, 9 orders, 15 genera, and 35 taxa (S1 Table). The 138 pyrophilous bacterial isolates comprised 17 genera and 71 taxa whereas the 152 pyrophilous fungal isolates comprised 8 genera and 30 taxa (S2 Table).

**Fig 2:**
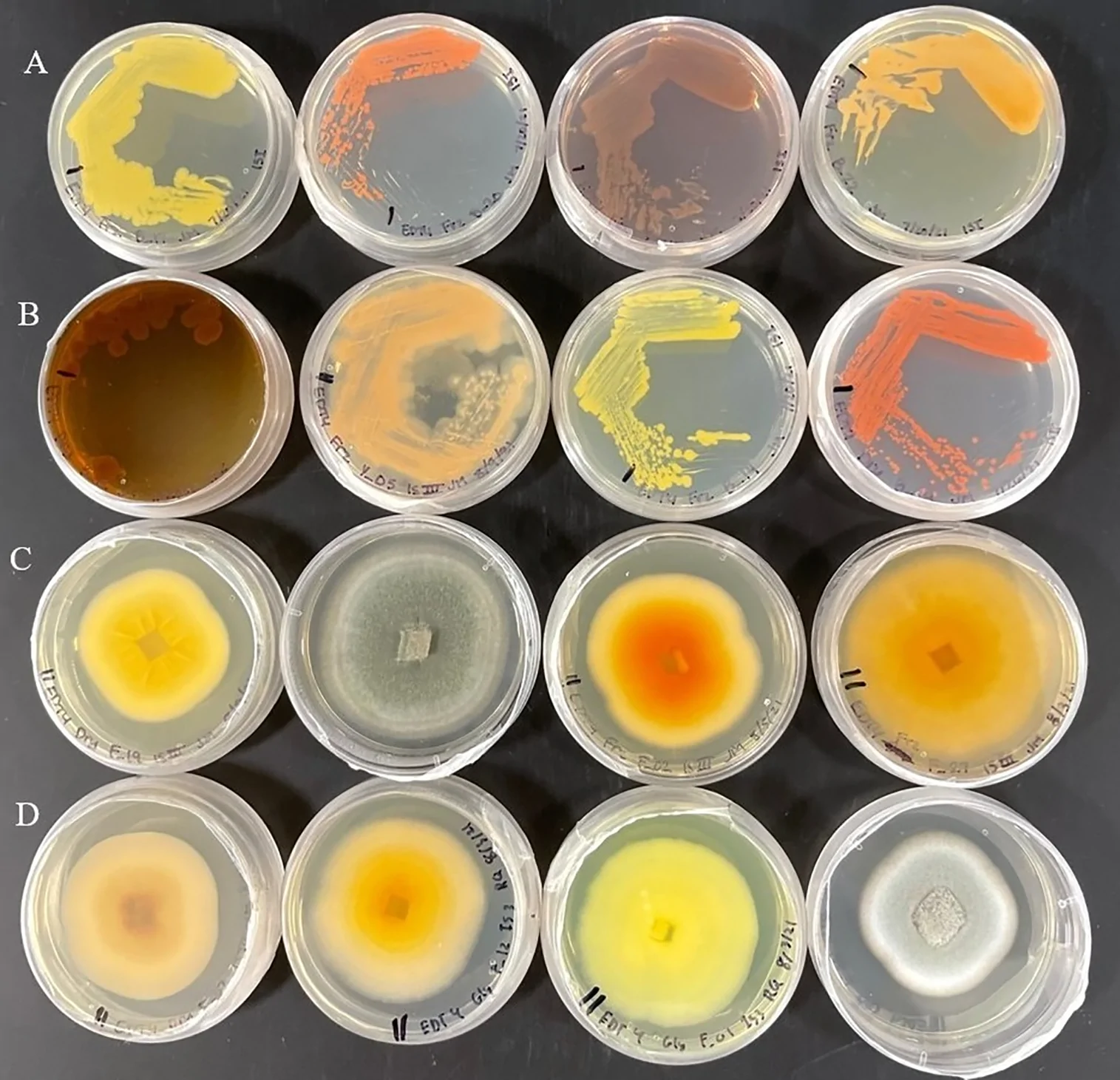
A snapshot of the morphological diversity of the cultures obtained from the El Dorado Fire across all soil storage and media type experiments. Identities were obtained via Sanger sequencing of the 16S or ITS regions for bacteria and fungi, respectively. Moving left to right across each row, the isolates shown are A)*Glutamicibacter bergerei, Arthrobacter agilis, Streptomyces mauvecolor, Kocuria rosea,* B) *Arthrobacter bussei, Holtermaniella festucosa, Pedobacter panaciterrae, Curtobacterium oceanosedimentum,* C) *Penicillium radiatolobatum, Penicillium fagi, Penicillium glabrum, Penicillium thomii,* D) *Penicillium chalabudae, Penicillium radiatolobatum, Penicillium murcianum, Penicillium adametzii.*

### Pyrophilous culture collection

The insights this study provided helped to guide our related work of creating a large and diverse collection of pyrophilous microorganisms of 544 isolates (286 bacteria; 258 fungi) from burned soils, mushrooms, and smoke derived from 7 California wildfires that burned from 2018 to 2022 (S3 Table). For bacteria, our 286 isolates represented 8 phyla, 16 orders, 35 genera, and 76 taxa. For fungi, our 258 isolates represent 3 phyla, 11 orders, 13 genera, and 34 taxa. The full table includes the fires of origin, collection times, substrate cultures derived from, isolate names, FASTA sequences, and BLAST summary statistics (S3 Table). We have begun to characterize a subset of these bacterial [52] and fungal [3] isolates with genomics, transcriptomics and biophysical assays.

Effect of Storage Method Selection on CFUs

Soil storage method significantly impacted the number of CFUs obtained per gram soil for both bacteria (ANOVA: F_1,4_ = 19.97, p < 0.001; Fig 3A) and fungi (ANOVA: F_1,4_ = 19.78, p < 0.001; Fig 3B). For both bacteria and fungi, soil stored air-dried (Bacteria: 2.31x10^5^ CFUs/gram, Fungi: 1.33x10^4^ CFUs/gram) and stored at −80ºC with glycerol (Bacteria: 1.31x10^5^ CFUs/gram, Fungi: 6.69x10^3^ CFUs/gram) retained the fewest average CFUs/gram soil. For bacteria, soils stored at 4ºC (6.98x10^5^ CFUs/gram) or at −80ºC without glycerol (6.39x10^5^ CFUs/gram) were not significantly different from fresh soils (5.73x10^5^ CFUs). For fungi, fresh soils had the highest average CFUs/gram soil (3.07x10^4^ CFUs/gram), followed by soil frozen at −80ºC without glycerol (2.66x10^4^ CFUs/gram), and then stored at 4ºC (2.16x10^4^ CFUs/gram).

**Fig 3:**
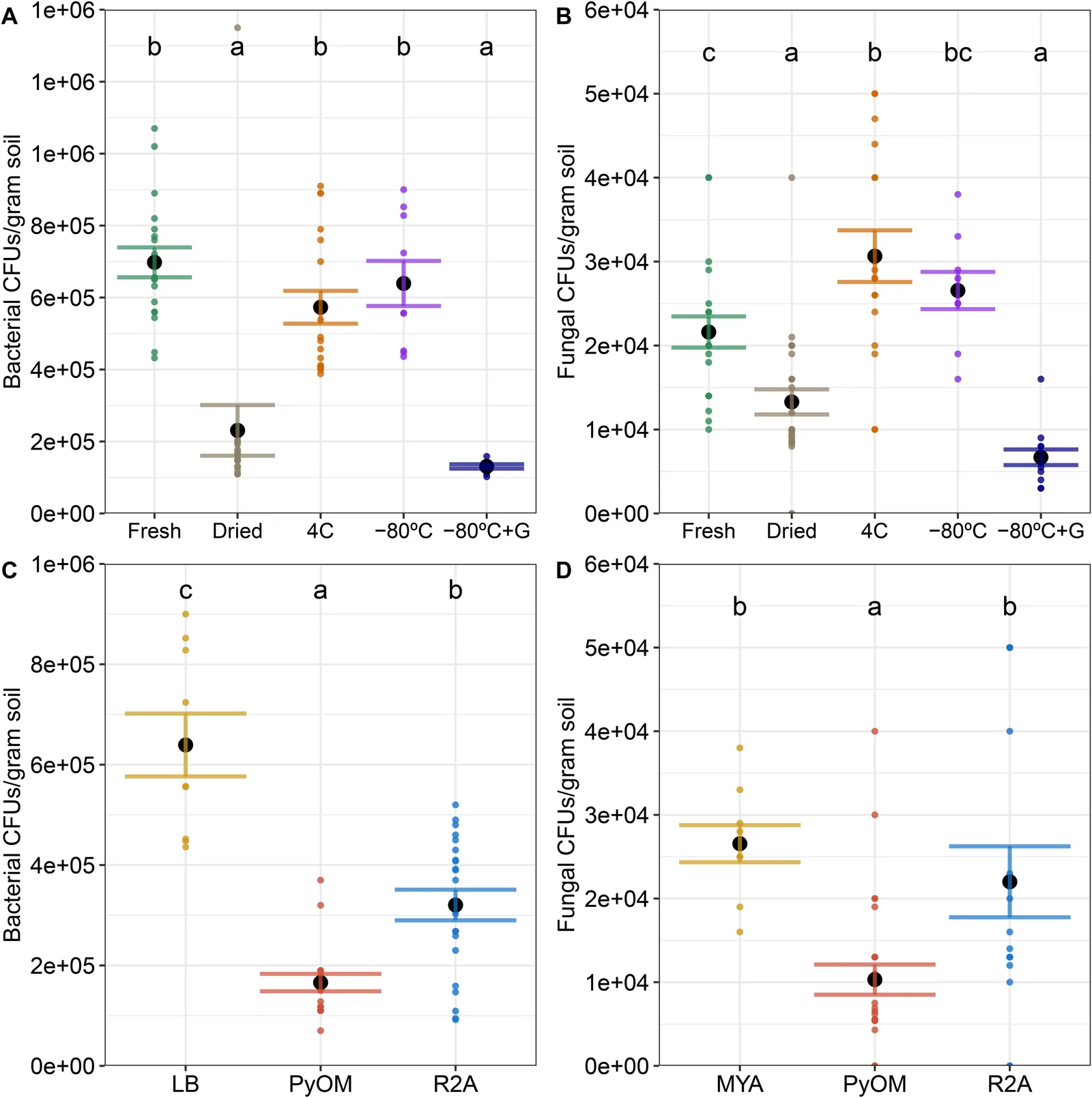
Calculated capturable CFUs per gram soil for A) bacteria and B) fungi among the tested soil storage methods and C) bacteria and D) fungi across tested media types. Points and bars are colored by treatment with bars indicating standard error of themean and black points indicating the mean CFUs per gram soil for each treatment. Significance was tested using ANOVA and Tukey Post-Hoc lettering has been applied. LB = Lysogeny Broth; PyOM = Pyrogenic Organic Matter; R2A = Reasoner’s 2 Agar, and G stands for glycerol.

### Effect of media selection on CFUs

Media type significantly impacted the number of CFUs obtained per gram of fresh soil for both bacteria (ANOVA: F_1,2_ = 39.43, p < 0.001; Fig 3C) and fungi (F_1,2_ = 7.77, p < 0.01; Fig 3D). For both bacteria and fungi, PyOM media yielded the fewest average CFUs per gram (Bacteria: 1.66x10^5^; Fungi: 1.03x10^4^), and rich media (LB for bacteria and MYA for fungi) had the highest average CFUs per gram (Bacteria: 5.73x10^5^; Fungi: 3.07x10^4^). R2A media had the second highest per gram average CFU counts for bacteria (3.20x10^5^) and for fungi, CFU counts (2.20x10^4^) were statistically equivalent to MYA.

### Effect of storage method selection on bacterial diversity

Soil storage method strongly affected richness of bacterial genera with fresh soil retaining the highest total (Fig 4A) and frozen soil retaining the highest pyrophilous richness (Fig 4B). Dried soil retained the lowest richness of bacterial genera among both total (Fig 4A) and pyrophilous isolates (Fig 4B). Across all soil storage treatments, we cultured a total of 21 bacterial genera and 64 taxa, and we found literature support to classify 13 genera and 51 taxa as pyrophilous or putatively pyrophilous (Fig 6A, S2 Fig in S1 File). Considering total bacterial taxa, fresh and frozen soil were equally rich (62% of genera), followed by soil frozen with glycerol (57% of genera), 4°C soil (48% of genera) with dried soil as least rich (38% of genera). Four genera (*Arthrobacter, Curtobacterium, Pseudarthrobacter, Streptomyces*) were isolated across all 5 storage treatments whereas 7 genera (*Brevibacterium, Fontibacillus, Glutamicibacter, Massilia, Ornithinimicrobium, Paeniglutamicibacter,* an unidentified Firmicute) were isolated from a single treatment (Fig 6A). Finally, 64% of taxa were isolated one time with only one taxon best identified as *Curtobacterium oceanosedimentum* observed in all storage treatments (S2B Fig in S1 File). Among the pyrophilous subset, frozen soil was the most rich (85% of genera), followed by fresh soil (62% of genera), soil frozen with glycerol (54% of genera), 4°C soil (54% of genera), and dried soil as least rich (38% of genera).

**Fig 4:**
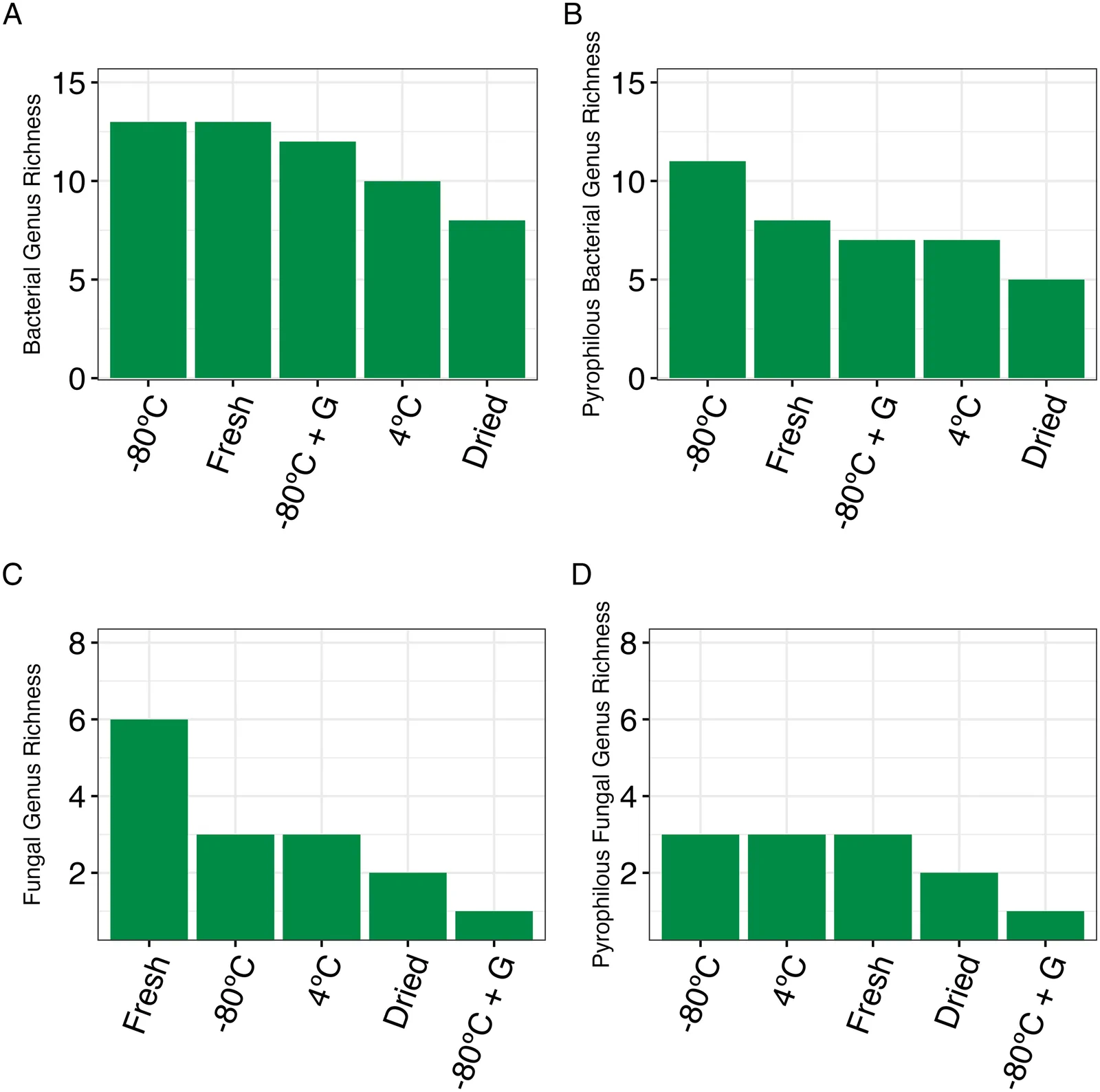
Summarized counts of A) total bacterial, B) pyrophilous bacterial, C) total fungal, and D) pyrophilous fungal genera across four soil storage methods compared to fresh soil when isolated on LB for bacteria or MYA for fungi. Treatments are ordered by highest amount of genera. G = glycerol.

### Effect of storage method selection on fungal diversity

Soil storage method strongly affected fungal richness with fresh soil retaining the highest total genera (Fig 4C) and fresh, frozen and refrigerated retaining equal numbers of pyrophilous genera (Fig 4D). Soil frozen with glycerol retained the lowest richness of fungal genera among total (Fig 4C) and pyrophilous isolates (Fig 4D). Across all storage treatments, we cultured a total of 6 fungal genera and 23 fungal taxa, of which we found literature support as pyrophilous for 3 genera and 20 taxa (Fig 6B, S3 Fig in S1 File). For total fungi, fresh soil was the most rich, capturing 100% of all 6 genera (*Aspergillus, Holtermanniella, Ochrocladiosporium*, *Penicillium*, and 2 uncultured Helotiales), followed by frozen and refrigerated soil capturing 50% of the genera, and 30% in dried soil. Soil frozen with glycerol performed the worst, capturing only *Penicillium* (17% of the total). For pyrophilous fungi, fresh, frozen, and refrigerated soil all captured 100% of the 3 genera (*Aspergillus, Holtermanniella, Penicillium*), whereas dried captured 2 (*Aspergillus, Penicillium*) and frozen with glycerol only captured 1 (*Pencillium*; Fig 6B).

### Effect of media selection on bacterial diversity

Media type strongly affected bacterial richness with R2A and LB both capturing the highest numbers of total bacterial genera (Fig 5A), R2A yielding the highest richness of pyrophilous genera (Fig 5B), and PyOM capturing the fewest total and pyrophilous bacterial genera. Across all media types from fresh soil, we cultured 23 total bacterial genera, 61 total bacterial taxa, and 16 pyrophilous bacterial genera and 49 pyrophilous bacterial taxa (Fig 6C; S4 Fig in S1 File). Among total bacteria, LB and R2A obtained equally rich communities (58% of genera), with the least in PyOM (33% of genera). Among pyrophilous bacteria, R2A captured the greatest richness (69% of genera), followed by LB (56% of genera) with PyOM yielding the lowest richness (44% of genera). Although PyOM media yielded the lowest diversity, it was the only media to isolate some important pyrophilous bacterial genera like *Noviherbaspirillum* and *Peribacillus* and interestingly, R2A was the only media to capture *Massilia* (Fig 6C).

**Fig 5:**
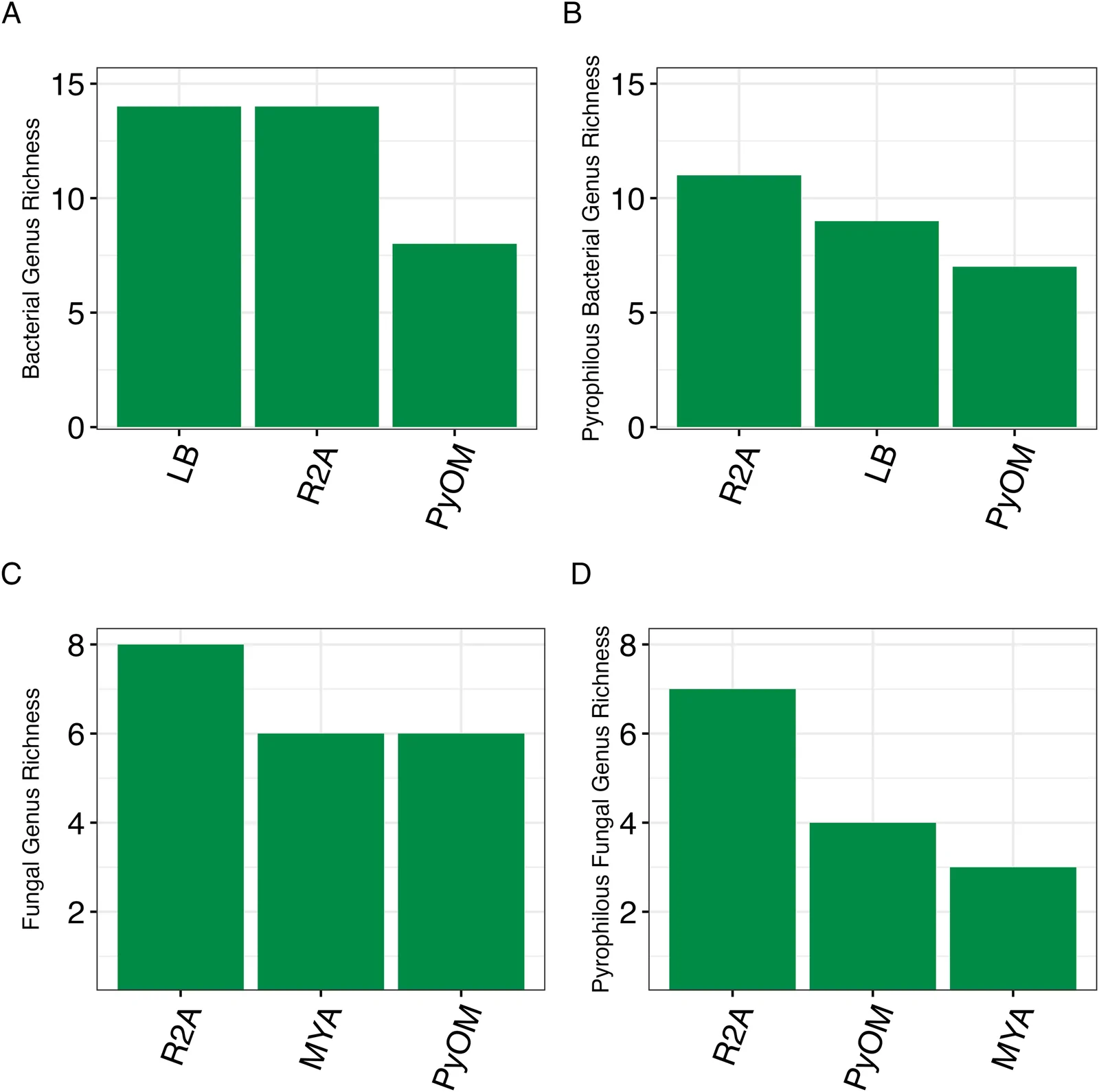
Summarized counts of A) total bacterial, B) pyrophilous bacterial, C) total fungal, and D) pyrophilous fungal genera (green) obtained from fresh soil tested across three media types. Treatments are ordered by highest number of genera. LB = Lysogeny Broth, MYA = Malt Yeast Agar, R2A = Reasoner’s 2 Agar, PyOM = Pyrogenic Organic Matter.

**Fig 6:**
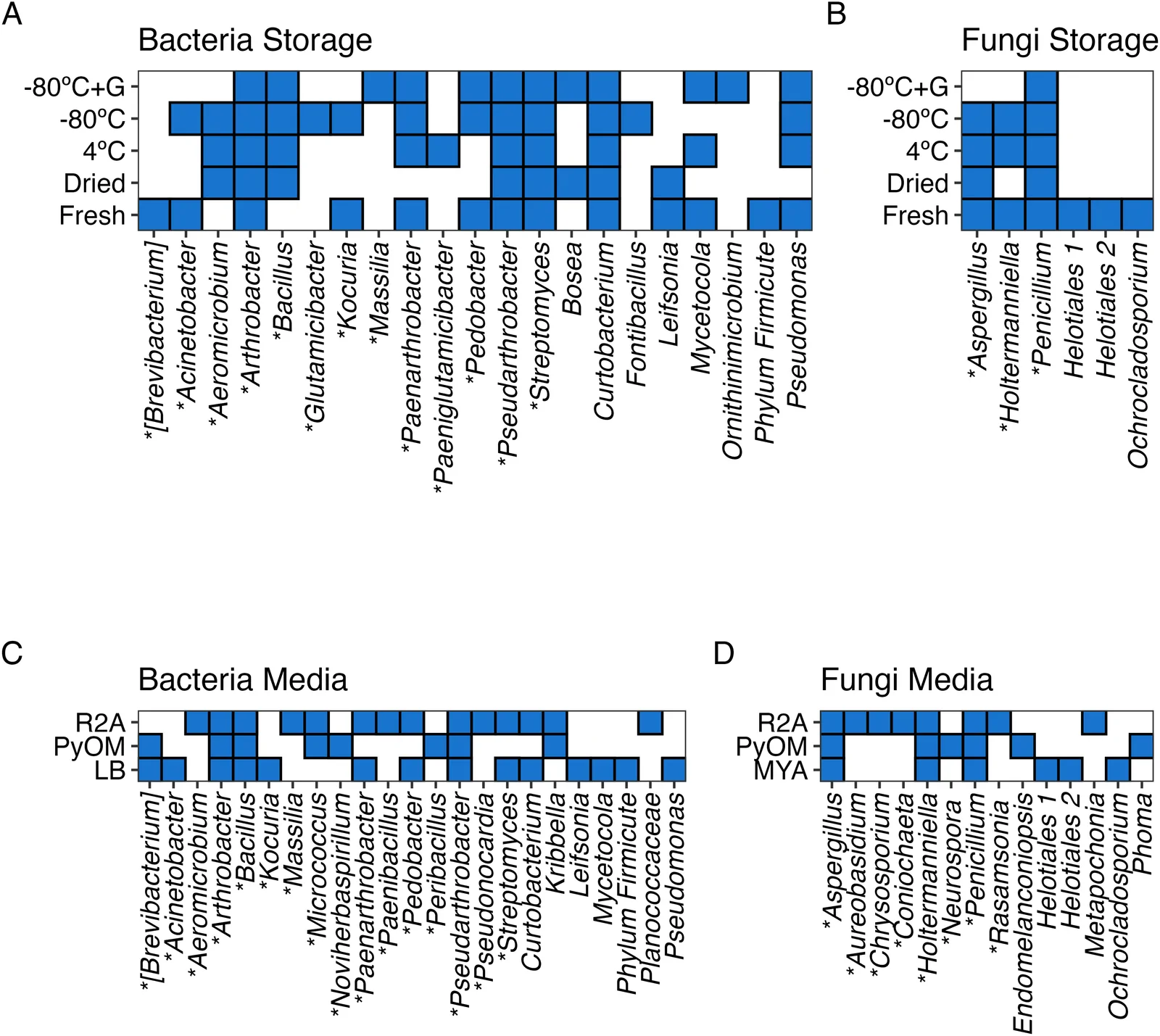
Heatmap-style visualization showing the diversity of A) bacterial genera and B) fungal genera across the soil storage experiment and C) bacterial genera and D) fungal genera across the media type experiment. Filled blue squares indicate the presence of the genus on the x axis in the treatment on the y axis. G = glycerol. LB = Lysogeny Broth, MYA = Malt Yeast Agar, R2A = Reasoner’s 2 Agar, PyOM = Pyrogenic Organic Matter Media. Asterisk (*) indicates that the taxa are pyrophilous or putatively pyrophilous.

### Effect of media selection on fungal diversity

Media type affected fungal richness with R2A capturing the most total (Fig 5C) and pyrophilous (Fig 5D) fungal genera whereas MYA captured the fewest. Across all media types from fresh soil, for total fungi we cultured 14 genera, and 29 taxa, and 8 genera and 23 taxa for pyrophilous fungi (Fig 6D, S5 Fig in S1 File). Among total fungi, R2A media captured the highest richness (57% of genera) while both MYA and PyOM were equally diverse (43% of genera). Among pyrophilous fungi, R2A also captured the highest richness (89% of genera), followed by PyOM (50% of genera), and MYA was the least rich (38% of genera). While PyOM did not capture the most diversity, it was the only media to capture *Neurospora* (Fig 6C), a well-documented model organism and pyrophilous fungus with relatively little knowledge about its ecology [53].

## Discussion

Here, we present results of an experiment to test soil storage and media selection to obtain the best diversity and abundance of bacterial and fungal isolates, which have informed our continual culturing efforts resulting in a collection of 544 pyrophilous microbes (286 bacteria; 258 fungi) (S3 Table). To our knowledge, this is the first study examining the impact of soil storage on fungal diversity, and the first large-scale culturing effort of pyrophilous bacteria, and among the largest soil and mushroom based culturing efforts of pyrophilous fungi since 1910 [54]. For both bacteria and fungi, freezing soil without glycerol yielded the highest number of pyrophilous genera on rich media, and from fresh soil, R2A yielded the most genera. Although it did not capture the highest number of CFUs or highest number of taxa, novel solid agar media we created from field-harvested PyOM was necessary to capture some key pyrophilous genera of interest [3,22,39,45,55] like *Neurospora* for fungi and *Noviherbaspirillium* for bacteria.

For bacteria, we conclude that freezing soil at −80°C is the best storage method relative to fresh soil, yielding the highest number of CFUs and total and pyrophilous bacterial genera when plated onto rich media. While CFU based analyses are useful in giving an approximation of the total number of culturable cells present in the original sample and a broad overview of the effectiveness of certain culturing practices at cultivating cells, CFUs do not capture the important distinction of isolate diversity. However, in this case, freezing soils without glycerol captured both the highest number of CFUs and yielded the vast majority of interesting pyrophilous bacterial genera on the post-fire bacterial succession literature [21,22,39,45–50,56]. While culturing from fresh soil did capture the greatest number of total genera, freezing soil captured the greatest richness of pyrophilous genera (Fig 4). Interestingly, freezing soil resulted in more total and pyrophilous bacterial genera being isolated, but adding glycerol increased the total number of taxa, and was necessary to isolate *Massilia*, which has been repeatedly observed post-fire [22,39,45,48,56], and we used genomic sequencing of this isolate to determined that it has many genes for PyOM degradation [52]. Recovery of certain taxa only after glycerol addition suggests heightened susceptibility to freezing-induced stress due to greater thermal sensitivity or reduced protection within aggregates and other microenvironments, indicating that freezing soils both with and without glycerol may improve recovery of these vulnerable taxa. Contrary to expectations based on the dry conditions of post-fire environments, dried soils yielded the lowest total and pyrophilous bacterial diversity. Although comparable studies are scarce, our findings contrast with a previous study reporting that refrigeration at 4°C better preserved microbial biomass and respiration than freezing at −20°C in unburned grassland and forest soils [32]. This discrepancy may reflect differences in soil structure and texture between those soils and our burned chaparral soils, which could influence microbial activity and the response of soil microenvironments to storage [57]. Although this study was based on 16S sequencing and not cultivation, a study examining impacts of storage time (0–14 days) and temperature (room temp, 4°C, and −20°C) similarly found that freezing at −20ºC preserved the most bacterial diversity, though they did not test freezing to −80°C [33], which may have decreased the taxa retained [58].

Soil storage method influenced pyrophilous fungi less than bacteria, with freezing at −80°C with glycerol performing worst, followed by air-dried, and all other methods performed similarly (Figs 4D, 6). Our hypothesis that air-drying would be most effective storage method was not supported; it instead produced the lowest CFU counts and microbial diversity, despite its common use in laboratory preparation [33,59]. The remaining methods (fresh, 4 °C, −80°C) might have performed similarly due to the high resilience of fungal resistant propagules like spores and sclerotia [60–62]. However, it is also possible that MYA may not be optimal for pyrophilous fungi, given the success of R2A and PyOM media (Figs 5D, 6D), and repeating storage experiments with these media may improve fungal isolate recovery. Notably, we know of no other studies that have examined how soil storage affects fungal cultivation, highlighting the need for cross-kingdom testing of these methods.

The results from the media type experiment showed that R2A produced the most diverse pyrophilous bacterial (Fig 5B) and fungal (Fig 5D) isolates. In addition, R2A produced the second highest number of CFUs after the rich medias for bacteria (LB) and fungi (MYA) (Fig 3). Because R2A was developed to isolate oligotrophic potable water bacteria [63], its success suggests that many pyrophilous microbes oligotrophic conditions. Although microbial life history strategies under stress are highly variable and nuanced [64], oligotrophic bacteria and fungi may be favored under increasing stress gradients (e.g., carbon, moisture, or heat stress) [65], making R2A well suited to capturing diverse microbes from post-fire environments. However, this is surprising because post-fire environments are often enriched in nitrogen and phosphorus despite major changes in carbon availability [28,34]. Pyrophilous taxa may therefore be less influenced by elevated nutrients and instead reflect the broader pattern of oligotrophy under environmental stress. As such, it is possible that availability of certain carbon forms plays a more integral role in post-fire nutrient acquisition and should be studied further. Although prior studies have explored media effects on cultivability [17,31,66], they often optimize single media, use CFU-only metrics, or rely on rich media. Broader investigation of media conditions is needed to improve cultivation, and our findings may guide future media development.

Finally, although our home-made PyOM media captured the lowest CFUs of bacteria and fungi, and the lowest richness of bacterial genera, it captured the second highest fungal richness and was necessary to isolate key bacterial (i.e., *Noviherbaspirillum soli*) and fungal (i.e., *Neurospora*) pyrophilous taxa of interest. Although we may have missed some pyrophilous microbial taxa because we use a water-based extraction to create our PyOM media, which might have precluded key nutrients required for post-fire metabolism that are less water-soluble in PyOM [67], here, isolating finicky pyrophilous taxa of interest was improved by the low-density growth we observed in PyOM. Rich media like LB, MYA, and potato dextrose agar are often plagued by fast growing ruderal species that can outpace taxa that grow at lower density [68].

The *Noviherbaspirillum soli* we isolated is particularly interesting since it has plasmids with genes for PyOM degradation that have been moved across bacterial orders and cross-kingdom to a pyrophilous fungus that we also isolated in this experiment [3,52]. *Noviherbaspirillum soli* has also been observed as highly dominant in many post-fire systems [22,24,39,45]. Likewise, *Neurospora* famously has thermotolerant spores [69], fruits abundantly on burned bark [55], and is widely used as a genetics model organism despite little understanding about its autoecology [53]. Therefore, although R2A maximizes pyrophilous bacterial and fungal diversity, and is also easy to replicate and readily available, including PyOM media is recommended for isolating the most finicky and interesting pyrophilous microbes.

While we made significant progress in evaluating culturing practices for environmental microbes, particularly pyrophilous bacteria and fungi, important limitations remain. CFU estimates were not normalized to dry soil mass to avoid potential loss of sensitive taxa during drying, limiting direct comparison with other studies. Except for the home-made PyOM media, we used commonly available laboratory media, and grew all isolates aerobically and at room temperature, despite the vast range of possible medias to use [66], and variations in growth temperatures [47], substrates [70], and pH [16], that may further enhance cultivation. Colony selection based on morphology introduces further bias, as morphologically similar species may have been grouped or missed [71,72], and dilution-to-extinction approaches may recover additional species-level diversity [73,74]. However, our approach was a practical compromise given the scale of isolation, preservation, and identification required. Nevertheless, the broad contrasts among rich, oligotrophic, and PyOM media provide useful insights into pyrophilous microbial cultivation. Our study was also designed as a broadly accessible foundation, thus we endeavored to use standard laboratory materials and protocols available to most research institutions, and accessible for undergraduates, which included most of our co-authors. Despite these caveats, we recovered a broad diversity of bacterial and fungal taxa (S1, S2 Tables) and observed clear, meaningful differences across media and storage conditions.

## Conclusions

Here, we present a systematic and large-scale test of how soil storage and media type affect bacterial and fungal cultivation from burned soils. We found that storing soils at −80°C without glycerol and combining rich media with R2A preserved the greatest diversity of culturable pyrophilous bacteria and fungi compared to fresh soils. However, certain pyrophilous taxa of interest were only captured from soil frozen with glycerol, like the bacteria genus *Massilia*, and on our novel PyOM media, like the fungus *Neurospora* and bacterial genus *Noviherbaspirillium*, indicating a potential value in using two media and storage types. Obtaining the cultures is the first step to characterizing their traits via ‘omics and phenotypic methods and sequencing genomes from isolates with concurrent biophysical assays is a powerful yet under-used tool to gain insight into microbial traits and connect genomic information to trait expression. Indeed, we have already used isolates from our pyrophilous culture collection to identify the genomic mechanisms underpinning broad capacity to degrade PyOM among pyrophilous bacteria [52] and identify trait trade-offs among pyrophilous fungi [3].

## Supporting information

S1 Table All isolates obtained across both experiments. For supplementary table S1, please see the Microsoft Excel spreadsheet titled “Table S1.xlsx”. Table S1 shows all isolates obtained from the El Dorado Fire 6 months post-fire across all experimental treatments.
(XLSX)

S2 Table All pyrophilous isolates obtained across both experiments. For supplementary table S2, please see the Microsoft Excel spreadsheet titled “Table S2.xlsx”. Table S2 shows all pyrophilous isolates obtained from the El Dorado Fire 6 months post-fire across all experimental treatments.
(XLSX)

S3 Table All isolates present in the Glassman Lab post-fire microbial culture collection. For supplementary table S3, please see the Microsoft Excel spreadsheet titled “Table S3.xlsx”. Table S3 shows all isolates obtained from all fire-related culturing efforts along with information on the substrate the isolates were cultured from and the relative fire information.
(XLSX)

S1 File Enrightetal supplementary materials PlosOne contains 5 supplementary figures. (DOCX)
