# Supplementary material for: Soil storage and growth media shape culture-based recovery of pyrophilous bacteria and fungi from burned soils
Source: PLoS One. 2026 Nov 13;21(11):e0359320. doi: 10.1371/journal.pone.0359320 (PMC13766225; doi:10.1371/journal.pone.0359320)
Supplement: Supplementary file 1 [file pone.0359320.s004.docx]

Soil storage and growth media shape culture-based recovery of pyrophilous bacteria and fungi from burned soils

**5 Supplementary figures**

**Figure S1**: A) Schematic diagram visualizing the vacuum siphoning set up for the creation of PyOM media. Prior to siphoning pyrogenic matter was ground, added to water, and heated at 40ºC overnight. After allowing 1 day of settling, the solution was vacuum siphoned as above. This extract would then be filtered through cheesecloth and coffee filters and allowed to settle one additional time before sterilization. B) And example of the field-sourced pyrogenic organic matter used to make PyOM media. C) An example plate of solid PyOM agar media.

**C**

**B**

**A**

**Figure S2:** Heatmap-like visualization of the diversity of all bacterial A) genera and B) species across all storage method treatments. Filled blue squares indicate the presence of the genus on the x axis in the treatment on the y axis. Asterisk (*) indicates that the taxa are pyrophilous or putatively pyrophilous. G = glycerol.

A) Bacterial Genera – Storage

B) Bacterial Species – Storage

**Figure S3**: Heatmap-like visualization of the diversity of all fungal A) genera and B) species across all storage method treatments. Filled blue squares indicate the presence of the genus on the x axis in the treatment on the y axis. Asterisk (*) indicates that the taxa are pyrophilous or putatively pyrophilous. G = glycerol.

A) Fungal Genera – Storage

B) Fungal Species – Storage

**Figure S4.** Heatmap-like visualization of the diversity of all bacterial A) genera and B) across all media type treatments. Filled blue squares indicate the presence of the genus on the x axis in the treatment on the y axis. Asterisk (*) indicates that the taxa are pyrophilous or putatively pyrophilous. LB = Lysogeny Broth, R2A = Reasoner’s 2 Agar, PyOM = Pyrogenic Organic Matter Media.

A) Bacterial Genera – Media

B) Bacterial Species – Media

**Figure S5.** Heatmap-like visualization of the diversity of all fungal A) genera and B) across all media type treatments. Filled blue squares indicate the presence of the genus on the x axis in the treatment on the y axis. Asterisk (*) indicates that the taxa are pyrophilous or putatively pyrophilous. MYA = Malt Yeast Agar, R2A = Reasoner’s 2 Agar, PyOM = Pyrogenic Organic Matter Media.

A) Fungal Genera – Media

B) Fungal Species – Media
